# Impact of Geriatric Admissions on Workload in the Emergency Department

**DOI:** 10.3390/healthcare11040593

**Published:** 2023-02-16

**Authors:** Tomasz Kłosiewicz, Monika Rozmarynowska, Patryk Konieczka, Mateusz Mazur

**Affiliations:** Chair of Emergency Medicine, Poznan University of Medical Sciences, 7 Rokietnicka Street, 60-608 Poznan, Poland

**Keywords:** emergency medicine, elderly, geriatric assessment, administration and organization, organizational efficiency, emergency hospital services

## Abstract

**Background**: Due to the increase in life expectancy, both the general population and the population of patients of emergency departments (ED) are getting older. An understanding of differences, workload and resource requirements may be helpful in improving patient care. The main goal of this study was to evaluate the reasons for geriatric admissions in the ED, identify typical medical problems and assess the number of resources in order to provide more effective management. **Methods**: We examined 35,720 elderly patients’ ED visits over the course of 3 years. The data collected included age, sex, timing and length of stay (LOS), use of various resources, endpoint (admission, discharge or death) and ICD-10 diagnoses. **Results**: The median age was 73 years [66–81], with more females (54.86%). There were 57.66% elderly (G1), 36.44% senile (G2) and 5.89% long-liver (G3) patients. There were more females in the older groups. The total admission rate was 37.89% (34.19% for G1, 42.21% for G2 and 47.33% for G3). The average length of the patient’s stay was 150 min [81–245] (G3 180 min [108–277], G2 (162 min [92–261]) and G1 139 min [71–230]). Heart failure, atrial fibrillation and hip fracture were the most common diagnoses. Nonspecific diagnoses were common in all groups. **Conclusion**: The vast majority of geriatric patients required considerable resources. With increasing ages, the number of women, LOS and number of admissions increased.

## 1. Introduction

Due to the ongoing extension of human life, the civilizational progress and the improvement of the quality of life throughout the world, the percentage of people in a post-working age is systematically growing. This trend is visible especially in highly developed countries. In 2010, around 8% of the world’s population was 65 years or over, and this is expected to double to 16% by 2050 [1]. According to the even more troubling demographic forecasts of the General Statistical Office of Poland, in 2050, the share of people of working age in the population will amount to 57%, with as much as 32.7% aged over 65, and less than 11% of those in a pre-working age [2]. This ageing process of the Polish population will cause a significant impact on healthcare, including hospital emergency departments (ED). The percentage of elderly people presenting to EDs has been increasing yearly, from 23.5% in 2017 to 27.7% in 2021 [3,4].

As much as 75% of older people with a pre-existing chronic disease such as advanced heart failure, lung diseases or cancer attend an ED in the last 6 months of their lives [5]. Other authors strictly suggested that age is undervalued as a risk factor for emergency hospital admissions [6]. Such a visit may become a turning point, determining the course of further treatments for this group of patients. Older adults discharged from the ED are more likely to experience difficulties in everyday functioning and a reduction in their quality of life [7]. Moreover, due to physical disability, seniors have more difficulties accessing primary health care. Seniors, as a group with special needs, require more staff involvement. Moreover, geriatric patients may pose a challenge to medical staff because of the unusual symptomatology of many diseases. Additional ED resources and coordination of care, as well as a complex approach to the geriatric population in emergency care, brings a benefit to patients and healthcare systems and optimizes outcomes [8,9].

In Poland, the topic of ED care for seniors still is not very prevalent. So far, no national guidelines have been created to help identify and solve the main problems concerning this particular group of patients. According to Ukkonen, the number of ED visits in the geriatric population will more than double in the upcoming 20 years [10]. Therefore, we find it urgent to evaluate the general burden of ED geriatric visits and establish the baseline for further interventions. Furthermore, the knowledge and understanding of differences in age groups, workload and resource requirements in ED would allow caregivers to provide more effective management and improve care.

Therefore, the aim of this study was to evaluate the reasons and epidemiology of geriatric admissions in the ED and identify the major medical problems, as well as the related burdens to the healthcare providers.

## 2. Materials and Methods

### 2.1. Ethical Consideration 

According to Polish law, this study did not meet the criteria of a medical experiment and does not need the approval of the Bioethics Committee.

### 2.2. Emergency Department Organization 

The study was conducted in a Community Emergency Department at HCP Medical Centre located in the center of Poznan, Poland. This is one of four EDs in the Poznan area. The city and district of Poznan had a population of 936,000 in 2020. Our ED is visited by approximately 32,500–34,500 patients every year. Its typical personnel comprise emergency medicine doctors (both specialists and residents). The ED also employs doctors’ aides, as well as nurses and paramedics. The emergency department is a teaching center, both for students of the Poznan University of Medical Sciences and for Emergency Medicine Residents. There was neither a geriatric ward nor a geriatric specialist available in our hospital.

### 2.3. Data Collection

All the data used in this study were collected from the Hospital Information System used in our emergency department. Its database structure allowed us to filter data according to the set of rules set forward by the authors, that is, the age of patients above or equal to 60 years of age at the time of the beginning of the hospital stay, and the date of the visit between April 2019 and March 2022 (3 full years of data collection—the total number of admissions was 97,669.). Those were the main inclusion criteria that were met by 36,001 individuals. A total of 281 were excluded due to a lack of required data. Therefore, 35,720 patients qualified for a further analysis. The collected data included age and sex as typical demographic indicators, as well as ICD-10 diagnosis, timing of the ED visit, including time of admission to the ED and total length of stay in the ED. Data have also been filtered in search of procedures performed on patients, including any medication administration (p.o., i.m., i.v. or nebulized) with sedatives, catecholamines and rapid sequence induction drugs, filtered separately. We also checked for oxygen therapy, Foley catheter insertion, blood transfusions, wound sutures, limb immobilizations and radiological scans (both plain X-rays and CT scans). We also determined the endpoint of the ED visit (discharge or admission). All the data were exported to data sheets, filtered and checked for flaws. Patients were divided into 3 subgroups according to the World Health Organisation guidelines: (1) youngest-old (elderly age, G1)—aged 60–74; (2) middle-old (senile age, G2)—aged 75–89; (3) oldest-old (long-livers, G3)—aged 90 and over.

### 2.4. Outcomes

Our primary outcomes were: (1) admission rate defined as percentage of patients referred to another hospital unit; (2) length of stay defined as the time between the first examination made by the physician and admission or discharge; (3) resources required during the diagnostic process or treatment (any laboratory tests, radiological scans, medications administered, urinary catheter, oxygen supplementation, suturing, cast immobilization, catecholamines’ infusion, rapid sequence induction and blood transfusion). All these parameters reflected the burden of the work of ED staff. Our secondary outcome was the evaluation of formulated diagnoses according to the International Classification of Diseases, 10th Revision (ICD-10).

### 2.5. Statistical Analysis

An analysis was performed using the Statistica 12 software (Tibco Inc., Tulsa, OK, USA). Descriptive statistics of measurable variables were performed. The categorical variables were expressed as numbers (n) with percentages (%). Quantitative data were first checked for normal distribution using the Shapiro–Wilk test. As they did not present a normal distribution, they were presented as median (interquartile range). To evaluate the significance of differences, the Kruskal–Wallis test followed by a post hoc test were used as appropriate. A value of *p* < 0.05 was considered statistically significant.

## 3. Results

### 3.1. Study Group

The total number of admissions in the ED in the study period was 97,669. The study population comprised of 36,001 patients aged 60 and above—36.86% of total ED visits. Two hundred and eighty-one reports were excluded due to the lack of basic data concerning diagnosis, time ranges, age or sex of the patient. Therefore, 35,720 individuals were qualified for a further analysis. 

There were 19,598 females (54.86%) and 16,122 males (45.14%). The median age was 73 years [66–81]. The oldest patient was 107 years old. 

There were 20,597 individuals (57.66%) in the elderly age (G1), 13,019 patients (36.44%) in the senile age (G2) and 2104 patients (5.89%) in the long-livers group (G3).

The percentage of the male gender in particular groups was as follows: G1 51.46%, n = 10,600; G2 38.02%, n = 4951; G3 27.13%, n = 571.

According to the triage category assigned, 648 patients (1.81%) were classified as needing immediate help (red), 5962 patients (16.69%) were classified as very urgent (orange), 16,614 patients (46.51%) were classified as urgent (yellow), 11,556 patients (32.35%) were classified as standard (green) and 940 patients (2.63%) were classified as non-urgent (blue).

### 3.2. Primary Outcomes

Admission rate in the whole group was 37.89% (n = 13,536). One hundred and seventy (0.48%) patients died in the ED. The remaining 22,014 (61.63%) individuals were discharged home. The median age of the admitted patients was 74 [67–83] years old, whereas for discharged patients it was 72 [66–83] years old. The difference was small but proved to be statistically significant (*p* < 0.001). The admission rate for particular subgroups was as follows: for G1—34.19%, for G2—42.21% and for G3—47.33%. The destination ward for admitted patients was defined in 10,635 cases. Details were presented in Table 1.

**Figure 1:**
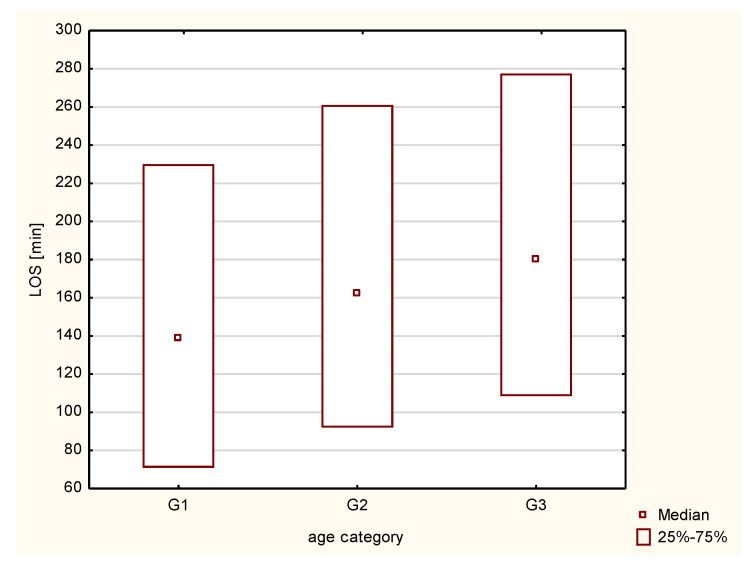
Graphical presentation of the length of stay in the emergency department in particular subgroups. G1—elderly age; G2—senile age; G3—long-livers; LOS—length of stay in the emergency department.

The median LOS was 150 min [81–245]. The shortest LOS was 5 min and the longest was 1438 min. The median LOS was the longest in the G3 subgroup (180 min [108–277]), then in the G2 (162 min [92–261]), with the shortest in the G1 group (139 min [71–230]). Differences between subgroups were statistically significant (*p* < 0.001 for all comparisons). The comparison of the LOS between subgroups was presented in Figure 1.

**Figure 2:**
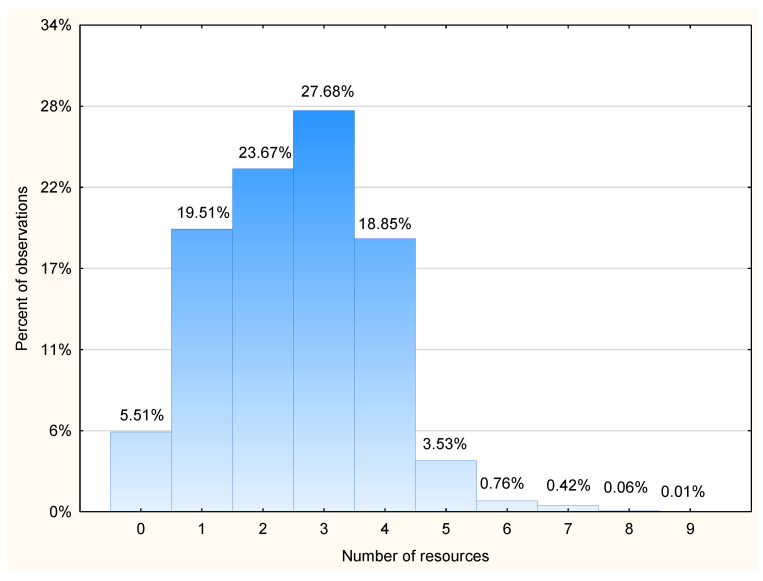
Percent of patients who required respective number of resources. Resources were defined as any laboratory tests, radiological scans, medications administered, urinary catheter, oxygen supplementation, suturing, cast immobilization, catecholamines infusion, rapid sequence induction and blood transfusion.

Almost all subjects had radiological diagnostics performed. More than half had laboratory tests performed. Moreover, more than half of the patients received medications. As much as 35.25% (n = 12,592) required continuous monitoring. The detailed number of resources used for patients was presented in Table 2. Fifty percent of patients required two or three resources. No resource was required in only 5.51% of cases (n = 1968), whereas 23.63% (n = 8439) required more than three resources. A detailed distribution was presented on Figure 2. 

### 3.3. Secondary Outcomes

There were a total of 766 different diagnoses stated at the endpoint. The analyzed groups differed in the number of the various diagnoses recorded (G1-666; G2-523; G3-244). The younger the group, the more heterogeneous were the diagnoses. 

Table 3 showed the 15 most common diagnoses in each subgroup. It can be noticed that in the first two groups, similar conditions were diagnosed. However, in G3, hip fracture, pneumonia and urinary tract infection were diagnosed far more frequently. Less precise diagnoses such as chest pain (R07), abdominal pain (R10), other symptoms and general signs (R68) were also more frequent in groups G1 and G2 than in G3.

Injuries were diagnosed in 7101 individuals (19.87%). Non-trauma patients comprised 80.12% (n = 28,619) patients. The highest percentage of trauma patients was in the G3 group (24.09%, n = 507), followed by the G1 group (20.08%, n = 4137). The least injuries occurred in the G2 group (18.87%, n = 2457). The most common injury was the fracture of the femur, open wound of the head and fracture of the forearm. 

## 4. Discussion

The purpose of this study was to evaluate the epidemiology of geriatric emergency admissions and the burden of work in the ED. According to the best of the authors’ knowledge, this was the first population-based study concerning geriatric challenges in the ED in Poland. A key element in improving the quality of care and reducing the mortality rate of seniors in the ED is the further development of the care system. Elderlies are a special group of patients requiring specific skills, tools and organizational models of emergency care on behalf of ED personnel to meet their complex needs.

ED becomes a place where key decisions concerning further healthcare are often made for this group of patients. One of the most important tasks of the ED physician is to identify indications for further hospitalization. This is an important issue because the mortality rate after hospitalization increases with patients’ age [11,12]. According to the results of our research, the frequency of hospitalization was much higher compared to younger groups. Other studies presented various results. In the research by La Manita et al., the admission rate was 65.4%, but they included only patients aged 75 and above [13]. In contrast, Lee and colleagues reported an admission rate of 40.3% [11]. These results are difficult to compare; however, Parker established the increasing age as one of the most predictive variables of the need for admission. This ratio depends largely on the organization of work and local considerations. In our opinion, the fact that there are emergency physicians working in the ED also influences the rate of admissions. It is possible to perform multiple procedures on seniors, such as, for example, a thoracocentesis on a patient with pleural fluid in the course of chronic heart failure. This helps to avoid unnecessary admissions. In countries with well-developed outpatient care, certain diseases can be treated and monitored in out-of-hospital settings, but this requires organized discharge planning, specialist follow-up, primary care physician involvement and disease management programs [14,15]. Those elements are still far from perfect in the Polish healthcare system.

In our group, there were 10% more women than men. We also noticed that in the older groups, the sex ratio changed in favor of females. Women have a longer life expectancy than men and therefore this result is not surprising. The most common diagnoses in our oldest group were those typical of females. Moreover, older women are more prone to complications related to heart disease [16].

For most patients admitted to the hospital, the target department was the Internal Medicine Ward and Cardiology Ward. These results are similar to those reported by Lee et al. [11]. The reasons for this condition can relate to changes taking place in the human body with age: multiple diseases, polypharmacy and hygiene throughout life, as well as socioeconomic factors. Hence, it is important to look at this group of patients in a special way and to prepare the ED staff substantively to work with them according to strictly defined guidelines tailored to their needs. It may be helpful to organize subacute care wards. These units are dedicated to individuals, who might benefit from a short period of inpatient stay within a less acute setting such as electrolyte disbalance. Moreover, consultant geriatrician-led teams performing a comprehensive geriatric assessment within the ED can reduce admission rates among older patients [17]. However, a major barrier for that in Poland is the lack of an adequate number of geriatric specialists. 

Longer LOS has been determined as a risk factor for developing delirium and an overall higher mortality [18,19]. Delirium prevention and treatment is a major challenge and the misdiagnosis rate is as high as 64.5% [20]. Our research has demonstrated that the duration of patients’ stay in the ED increases with age. This may be due to the fact that senior patients require a more thorough analysis of symptoms resulting from less common presentations as well as time-consuming consultations with various specialists. Biber et al. estimated that the LOS of elderly trauma patients was 35 min longer when compared with the younger group [21]. Many various strategies of reducing the LOS were discussed so far. The process of laboratory/radiology testing, the decision making and discharge procedures may be all accelerated. The initial evaluation by a medical student or non-trainee resident may be useful. Moreover, the quality and availability of protocols plays an important role [22,23,24]. Wallis suggested that the implementation of a nurse-led model of ED care focused on frail older adults, reduced the ED length of stay, total admission rate and a same cause readmission [25]. Another strategy that is helpful in reducing the LOS is a fast-track model. This is a process developed to manage patients with less serious or easy to diagnose conditions who can be treated and discharged or admitted more quickly in a separate pathway [26]. In the case of a patient whose condition will require hospital admission (e.g., hip fracture), procedures performed in the ED should be limited. Among many concepts, the one that is tailored to the local environment should be chosen.

The majority of our patients required two or three resources within the ED. Almost all required radiology and more than half had laboratory tests performed. There was also a large group of patients requiring four or more resources. Such involvement in the diagnostic process increases not only the personnel involvement in patient care, but also the costs of stay at the ED. The increased demand for resources also indicates that the patients required a more complex differential diagnosis or the administration of more medications. Therefore, a high resources demand rate determines the prudent use of fast-track strategies for geriatric patients. However, if this strategy is to be implemented, it should be based on clear procedures and staffed by experienced clinicians.

Our results demonstrated that many of our patients were discharged with a non-specific diagnosis. In this category, the prevailing diagnoses were: abdominal pain, chest pain and other general symptoms and signs. In the elderly age, non-specific diagnoses were the most common. In our opinion, the high number of non-specific diagnoses may indicate the need for a better education of emergency physicians in the field of geriatrics. This is the most important finding, as patients with non-specific diagnoses have a high risk of mortality [27]. In the senile age, heart failure was predominant. The long-livers group differed completely from the others in the profile of diagnoses. Respiratory and urinary tract infections as well as femur fractures were more dominant. In a similar study conducted in Finland, the authors also noted that infections were more common in the oldest patients [10]. In this group, however, hip fractures occurred in less than 3 percent of patients. In our study, it was almost twice as frequent, making this diagnosis far more important. In addition, the highest number of injuries overall was recorded in this group. This may reflect a lthe need for improvement system of home care for seniors, including prevention and social services. The risk of falling may be reduced by group and home-based exercise programs, and home safety interventions [28].

### Limitations

The authors are aware of several limitations. Firstly, this was a single-center study. The results are difficult to transpose to other hospitals, as they may have a completely different work model. More and more emergency medicine specialists are now appearing in Polish EDs. However, it is still very common to find physicians from other specialties working in EDs. Secondly, we have designed a descriptive study without a control group. This means that we cannot strictly compare the results and conclude differences. This is an important limitation; however, we believe that it would be unreliable to compare such diverse groups. We decided to focus only on three geriatric groups. The LOS was calculated as the time to a final decision. It means that some patients could spend more time in the ED waiting to be transferred by ED staff. We are aware that the presented subject needs further analysis. We hope that, despite these imperfections, this study will be a good baseline for future investigations.

## 5. Conclusions

The majority of geriatric admissions required considerable ED resources. An older age was associated with longer LOS, higher admission rate and more female patients. Diagnoses in ED differed between groups. In long-livers, the major medical problems were hip fractures and infections, whereas for elderly and senile age patients, the most common were cardiovascular disorders. Intensive efforts should be made to organize ED fast tracks especially for the oldest patients. Moreover, a better education for emergency physicians in the field of geriatrics is recommended.

## Figures and Tables

**Table 1:** The destination ward for admitted patients.

Ward	n	%
Internal medicine	3018	28.39
Cardiology	2275	21.39
Neurology and Stroke unit	1375	12.93
Surgery	1206	11.34
Orthopedics	771	7.25
Infectious diseases	496	4.66
Psychiatry	353	3.32
Intensive care unit	223	2.10
Other	918	8.63

**Table 2:** The detailed number of resources used for geriatric patients.

Resource	n	%
Radiology	32,695	91.53
Laboratory test	20,103	56.28
Medications	18,351	51.37
Monitoring	12,592	35.25
Sedation	1529	4.28
Cast	1225	3.43
Suturing	968	2.71
Oxygen supplementation	587	1.64
Urinary catheter	582	1.63
Rapid sequence induction	442	1.24
Catecholamine infusion	408	1.14
Blood transfusion	177	0.50

**Table 3:** Most common diagnoses in each subgroup. Nonspecific diagnoses are marked with asterix. G1—elderly age; G2—senile age; G3—long-livers; D64—Other anemias; I10—Essential (primary) hypertension; I21—Acute myocardial infarction; I48—Atrial fibrillation and flutter; I50—Heart failure; I63—Cerebral infarction; J18—Pneumonia, organism unspecified; N39—Other disorders of urinary system; R07—Pain in throat and chest; R10—Abdominal and pelvic pain; R55—Syncope and collapse; R68—Other general symptoms and signs; S00—Superficial injury of scalp; S01—Open wound of head; S52—Fracture of forearm; S72—Fracture of femur; U07—COVID-19; Z03—Medical observation and evaluation for suspected diseases and conditions.

G1			G2			G3		
ICD-10 Code	n	%	ICD-10 Code	n	%	ICD-10 Code	n	%
R07 *	1074	5.21	I50	554	4.26	I50	123	5.85
R10 *	973	4.72	R10 *	527	4.05	S72	105	4.99
I48	635	3.08	R07 *	523	4.02	R10 *	75	3.56
R68 *	606	2.94	I48	488	3.75	R55 *	72	3.42
I10	530	2.57	R55 *	403	3.10	J18	70	3.33
I50	512	2.49	R68 *	327	2.51	N39	59	2.80
R55 *	464	2.25	I10	325	2.50	D64	58	2.76
U07	419	2.03	U07	309	2.37	R68 *	56	2.66
I21	415	2.01	S72	304	2.34	U07	54	2.57
Z03 *	374	1.82	D64	300	2.30	S01	54	2.57
S52	364	1.77	N39	299	2.30	I48	54	2.57
R06 *	349	1.69	I63	262	2.01	R07 *	47	2.23
I63	343	1.67	J18	241	1.85	S00	47	2.23
S01	342	1.66	I21	240	1.84	I63	43	2.04
N39	342	1.66	R06 *	229	1.76	R06 *	40	1.90

## Data Availability

All the data are available from the corresponding author on reasonable request.
